# A novel smartphone application for the informal caregivers of cancer patients: Usability study

**DOI:** 10.1371/journal.pdig.0000173

**Published:** 2023-03-03

**Authors:** Ingrid Oakley-Girvan, Reem Yunis, Stephanie J. Fonda, Elad Neeman, Raymond Liu, Sara Aghaee, Maya E. Ramsey, Ai Kubo, Sharon W. Davis

**Affiliations:** 1 Medable Inc., Palo Alto, California, United States of America; 2 Estenda Solutions, Inc, Wayne, Pennsylvania, United States of America; 3 Kaiser Permanente Northern California, San Francisco Medical Center, San Francisco, California, United States of America; 4 Kaiser Permanente Northern California, Division of Research, Oakland, California, United States of America; University of New South Wales, AUSTRALIA

## Abstract

Informal caregivers are a critical source of support for cancer patients. However, their perspectives are not routinely collected, despite health impacts related to the burden of caregiving. We created the TOGETHERCare smartphone application (app) to collect observer-reported outcomes regarding the cancer patient’s health and caregiver’s perceptions of their own mental and physical health, and to provide tips and resources for self-care and patient care. We enrolled 54 caregivers between October 2020 and March 2021 from Kaiser Permanente Northern California (KPNC), an integrated healthcare system. Fifty caregivers used the app for approximately 28 days. Usability and acceptability were assessed using questions from the Mobile App Rating Scale (MARS), the System Usability Scale (SUS), the Net Promoter Score (NPS), and semi-structured interviews. The caregivers’ mean age was 54.4 years, 38% were female and 36% were non-White. The SUS total mean score was 83.4 (SD = 14.2), for a percentile rank of 90–95 (“excellent”). Median MARS responses to the functionality questions were also high. The NPS score of 30 at the end of the study indicated that most caregivers would recommend the app. Themes from semi-structured interviews were consistent across the study period and indicated that the app was easy to use and helpful. Caregivers indicated a need for feedback from the app, suggested some changes to the wording of questions, the app’s visuals, and timing of notifications. This study demonstrated that caregivers are willing to complete frequent surveys about themselves and their patients. The app is unique because it provides a remote method to collect caregivers’ observations about the patient that may be useful for clinical care. To our knowledge, TOGETHERCare is the first mobile app developed specifically to capture adult cancer patient symptoms from the informal caregiver’s perspective. Future research will examine whether use of this app can help improve patient outcomes.

## Author summary

Family members and friends taking care of their loved ones who have been diagnosed with cancer often experience anxiety and depression due to the caregiving burden. Being able to report symptoms the loved one experiences as well as their own symptoms may provide health benefits to both the cancer patient and the caregiver. We created a smartphone app for informal caregivers of cancer patients (TOGETHERCare) to collect this information and to provide resources for self and patient care. Over 28 days, we tested usability of the application with caregivers of patients at Kaiser Permanente Northern California. The application included three surveys for caregivers to rate ease of learning and use. The average score on the three different rating scales was high and considered “excellent” compared with scores from other applications. Comments that caregivers provided during interviews also indicated that they found it easy to use and helpful. This study documents that caregivers are willing to complete frequent surveys about their loved ones and themselves. Based on the findings presented here and in other manuscripts, we hope to do further research to determine whether this app can help improve patient outcomes.

## Introduction

There is an urgent need to support informal caregivers of cancer patients. The number of individuals living with cancer has increased from 14 million in 2012 to an estimated 18 million in 2022 [1], and the number of informal caregivers has grown to 6.1 million [2]. Informal caregivers attend medical visits with and advocate for the patient, keeping track of physician instructions and medication changes, providing emotional support, and assisting with activities such as medication administration, wound care, transportation, meals, and finances [3]. Over half of informal caregivers have chronic health conditions of their own [4,5], and their health status can be worsened by the stress and demands of caregiving [6–16], which can then adversely affect their patient’s well-being [17–20]. For instance, overburdened informal caregivers seldom seek mental health care to deal with emotional distress [21–23], despite informal caregivers of patients with advanced cancer reporting more depression than the patients themselves [24]. Further, caregivers have little time to rest or engage in self-care behaviors (e.g., health check-ups or preventive screening), and they often fail to seek medical care when sick [25,26]. The negative effects of caregiving, including a higher mortality rate, are found to be greater among informal caregivers of patients with advanced cancer [24,27,28], particularly when patients are receiving treatments such as chemotherapy [29,30]. Despite the tremendous burden that informal caregivers frequently experience [24,27,28], their emotional and physical needs are often not supported. By developing a system where a mobile app can capture caregivers’ physical and mental health status that could be sent directly to their care providers, preventive interventions could be made before caregivers become severely ill. This has the potential to help the caregivers provide greater care for their loved ones, improving the quality of life and, thereby, prognosis of cancer patients.

Studies indicate that using an electronic symptom reporting system decreases caregivers’ emotional distress [31–33]. Given the high utilization of mobile technologies, mobile applications (apps) may provide a meaningful mechanism for symptom management reporting. Almost all (97%) Americans own a cell phone, and 85% own a smartphone [34]. Mobile phone health apps have been shown to improve self-management of health conditions and [35] increase health-promoting behaviors [36].

Previous research has shown that cancer survivor symptom self-reporting through patient-reported outcomes (PROs) increases patient health-related quality-of-life, decreases emergency room visits and hospitalizations, and increases survival [37–41]. Similarly, the caregiver can provide information and early warnings of declining patient health through observer-reported outcomes (ObsROs) [42–48]. The efficacy of ObsROs, however, has not been studied in the context of cancer, although healthcare providers believe they are important to their understanding of patients’ symptoms and responses to cancer treatments [49].

In this article, we introduce TOGETHERCare, a mobile health application (app) designed to monitor caregiver health, streamline ObsROs reporting, and increase access to resources on health and well-being. To the best of our knowledge, this app is the first to collect caregiver ObsROs about adult cancer patients undergoing intravenous chemotherapy or immunotherapy and report on the app’s usability and acceptability from the perspective of the caregivers.

## Methods

### Setting

This study was conducted with Kaiser Permanente Northern California (KPNC). KPNC is an integrated healthcare delivery system serving over 4.5 million members in Northern California.

### Study design

This single-arm, longitudinal, mixed-methods, prospective usability study included informal caregivers of cancer patients. We aimed to collect meaningful data to evaluate multiple parameters of usability including theme saturation in semi-structured interviews. We did not conduct any power calculations as this was not an intervention, so no effect size was anticipated.

To participate, both the cancer patient and their caregiver had to be eligible. Eligible cancer patients were adult (age 18 or older) KPNC members receiving intravenous chemo- or immunotherapy, living with a caregiver who was willing and eligible to participate, spoke English, and owned an iPhone 6 or above. Stage of cancer was not an inclusion or exclusion criterion. Cancer patients with serious mental health concerns or insufficient cognition to consent, as determined by their physician per KPNC policy, were not eligible to participate. Caregivers were identified by eligible patients based on who lived with them and spent the most time providing unpaid care. Eligible caregivers, lived with the patient, were 18 years of age or older, spoke English, and owned an iPhone 6 or above. There was no requirement for the length of time the caregiver lived with the patient. Patients and caregivers who owned an Android phone were ineligible because, at the time of the study, the application was available only on the iPhone. Caregivers were not required to be KPNC members.

After checking with the primary oncologists for contraindications to participation, study staff sent up to two recruitment emails, one week apart, containing brief study details and eligibility requirements to potential KPNC cancer patients. For those who expressed interest, the KPNC research associate screened for eligibility and reviewed study procedures with potential participants. The patient identified an interested caregiver who was then screened for eligibility. Recruitment was conducted between October 2020 and March 2021. We recruited 54 dyads (patient-caregiver pairs) as our objective was to collect complete study data from at least 45 caregivers.

After confirming eligibility, the research associate emailed the informed consent document and scheduled a call to review it. Informed consent was collected verbally and electronically in writing (eConsent) as one of the initial tasks provided through the app. Caregivers downloaded the TOGETHERCare app and were asked to use it at least several times per week for 28 days, during which the mobile app delivered specific surveys and activity requests at regular intervals (Table 1). Caregivers who completed the surveys received a $100 gift certificate. The KPNC Institutional Review Board (IRB) approved this study.

**Table 1:** Surveys included in the TOGETHERCare app.

Survey Title	Validated Instrument Name or Content Description	Frequency
*Caregiver Self-Assessments/Reported Outcomes (PROs)*		
Your Wellness	Patient-Reported Outcomes Measurement Information System (PROMIS) Global Health [50]	Weekly
Every Other Day	Sleep, exercise, fatigue, worry, missed medications	Every other day
Care Snapshot	Caregiving burden	Baseline, days 14 and 28
Quick Check-in	Financial and social distress, engagement and activation	Baseline and day 28
COVID-19 Your Input	COVID-19 knowledge, attitudes and behavior questions	Baseline and day 28
About You	Health literacy and demographics	Baseline
App Feedback	System Usability Scale (SUS) [51,52] Mobile App Rating Scale (MARS) [53] – 5 selected questions Net Promoter Score (NPS) [54]	Baseline, days 14 and 28
*Caregiver’s Assessments of Their Loved One (Patient) (ObsROs)*		
Loved One’s Short Symptom Report	National Cancer Institute Patient Reported Outcomes version of the Common Terminology Criteria for Adverse Events (PRO-CTCAE) [55] Eastern Cooperative Oncology Group performance status (ECOG) [56] Pre-appointment concerns	Weekly
Fast 4	Patient Reported Outcomes Measurement Information System (PROMIS) Item Bank v2.0 Physical Function–Short Form 4a [50]	Weekly
COVID-19 Patient Impact	COVID-19 impact on patient care	Baseline and day 28

### TOGETHERCare functions

The TOGETHERCare app was designed with input from caregivers, cancer patients, and healthcare providers as previously described [33]. The app included the following tabs: *Profile* for the study name and copy of the signed informed consent document; *Tasks* for informed consent, surveys related to caregivers’ health, caregiver demographics, and surveys related to patient health including a targeted symptom list and a pre-appointment concerns survey (Table 1); and *Resources* for information on how to contact the National Cancer Institute (NCI) and links to the Cancer Support Community and American Association for Retired Persons (AARP) webpages for caregivers. At the time of this study, the app did not include feedback or communications from clinicians regarding the users’ completed survey responses.

### Data collection

Usability, acceptability, and user satisfaction were collected through mobile app rating scales during the “App Feedback” task (Table 1) and semi-structured interviews.

#### Mobile app rating questions/scales

Three mobile app rating scales were collected at baseline, after approximately 14 days of use, and again at the end of the 28-day study period, via the app itself: a subset of questions from the MARS [53], the full SUS [51], and the NPS [54]. From the MARS, we selected five items that did not duplicate questions asked in the SUS. These questions covered functionality and visual appeal, with responses on a Likert scale of 1 (worst) to 5 (best) [53]. The functionality and aesthetics sections of the MARS have reliability coefficients of 0.87 and 0.90, respectively, and they correlate well with other validated instruments, which indicates good concurrent validity [57]. The SUS has 10 Likert-scaled items ranging from 1 (strongly agree) to 5 (strongly disagree). Eight items address usability and two address learnability. Total SUS scores range from 0 to 100, as do the scores for the usability and learnability sub-scales, with higher scores indicating better usability [52,58]. SUS scores are then converted to a percentile rank to show how the app rates relative to others [59]. The SUS has been used widely to study the usability of software and hardware, and a recent study shows that it has a similar distribution and interpretability when used to assess mobile health apps [60].

The NPS is derived by asking respondents to indicate the likelihood that they will recommend a product to a friend or colleague, using a 0 (least likely) to 10 (most likely) scale [54]. Respondents are defined as “promoters” of the product if they score 9–10, “detractors” if they score 0–6, and “passives” if they score 7–8. The percentage of detractors is subtracted from the percentage of promoters, giving the final NPS [61]. Although the usability questions were presented to the caregivers several times over the course of the study (Table 1), their responses were stable; thus, this article reports the summary statistics from the final survey instance (~28 days) only.

#### Semi-structured interviews

Two researchers (IOG, RY) conducted semi-structured interviews after 7 days of app use (with 54 caregivers) and after completion of the study (with 50 caregivers) after at least 28 days of use. Researchers interviewed all caregivers in the study, since coding and evaluation of thematic saturation were conducted at a later date and we could not go back to conduct interviews after the study ended. The semi-structured interview guide was developed through early testing with stakeholders [33] and by considering product development insight from our technology team. Caregivers were asked to elaborate their responses, and those responses prompted more questions. Prior to submission of the interview guides to the KPNC IRB, we were unable to find references related to qualitative analyses of caregiver applications designed to collect observer reported outcomes. Interview topics included anything confusing or useful about the app, thoughts about the frequency of the surveys in the app and the length of time it took to complete them, whether the app included information that the caregiver would like the patient’s support team to know, and whether there were other features the caregiver would like to see. In addition to these topics, the final interviews included outcome questions, presentation of planned revisions based upon earlier feedback, the NPS, and questions about possible additional features and their usefulness. The interviews were conducted and recorded for transcription using a secure video platform (Microsoft Teams). The KPNC Institutional Review Board specifically requested that no characteristics of the interviewees be included with the transcripts of the interviews, and to protect study subject confidentiality, that the ID numbers used for the quantitative data collection be altered in such a way that they could not be matched with semi-structured interview subjects.

#### Participant background characteristics

Demographics including age, gender, race, ethnicity, education, employment status, relationship to patient, and cancer health literacy were collected through the “About You” task in the app. Cancer health literacy was collected using the Cancer Health Literacy Test-6 (CHLT-6) [62].

### Analyses

Descriptive statistics were used to report the caregivers’ background characteristics and responses to the final usability survey questions collected via the app. For the analysis of the semi-structured interviews, we used content analysis to identify a set of major themes from the interviews. Content analysis is a methodology used for analyzing large amounts of textual data in order to discover underlying patterns as well as make inferences via a process of systematic categorizing and coding of the information [63,64]. This method and related similar methods have several precedents in the medical literature [65–67]. In order to glean insights from the data, the data are read in detail and initial codes are constructed by identifying and analyzing units of the data, which are then grouped into meaningful and related categories [68,69]. The unit of analysis may consist of words, sentences, paragraphs, or a section of ideas and thoughts [63]. In this case, we used all of these units, as the interviews were semi-structured in nature. Given the sample size, we did not attempt to quantify the frequency of the different categories and themes, as this may result in loss of context or confer spurious importance to a category, as frequent mentions may be due to willingness or ability to talk about a topic [64].

We followed the three main steps of inductive content analysis (data preparation, organization, and reporting), as recommended when there is limited knowledge about the phenomenon [70]. In data preparation, the researcher becomes deeply immersed in the data. After multiple readings of the textual data, the researcher decides on the unit of analysis, which can be words, sentences, or phrases. The organization phase involves open coding and creating categories, after which the categories are grouped or collapsed into higher order categories, and in the final phase of reporting, the meanings of the categories are described [63,70]. To ensure reliability, after a trained qualitative researcher (VS) completed the coding and theme-formation, a second researcher (YM) independently reviewed it. An additional member of the research team (SWD) reviewed discrepancies between the two researchers, and, with input from the lead researcher of the projects (IOG), achieved consensus.

The interview qualitative data and quotes presented are from the interviews that were coded up to the point we reached thematic saturation. After carefully analyzing and coding all Day 7 interviews and 18 of the final interviews conducted after Day 28, no new themes emerged as theme saturation had been reached, and thus no further coding for the remaining Day 28 interviews was conducted.

## Results

### Recruitment and retention

Recruitment emails were sent to 2,155 KPNC cancer patients. Among them, a total of 1,908 patients did not respond to the email or could not be reached by phone. Of the 247 patients, 166 were confirmed ineligible, 20 declined to participate before eligibility could be determined, and 7 declined to participate after learning more details about the study. Fifty-four patient-caregiver dyads were enrolled; 50 dyads completed all study requirements for a retention rate of 92.6%.

### Background characteristics of respondents

Of the 50 caregivers who completed the study, 62% (n = 31) were male and 38% (n = 19) female (**Table 2**). The majority (82%, n = 41) were not Hispanic or Latino, and 64% (n = 32) of the participants identified as White. Fifty-four percent (n = 27) had achieved a college degree, and nearly all (96.0%, n = 48) were cancer literate according to the Cancer Health Literacy Test Cancer-6 (CHLT-6) scores [62]. Each CHLT-6 response is either correct or incorrect; the mean number of correct responses for the six questions was 5.7, and the median was 6. Most of the patients (78%) attended by the caregivers were female, primarily due to the cancer type: breast (n = 17, 34%) and gynecological (n = 12, 24%). Gastrointestinal (n = 12), thoracic (n = 6), and other (n = 3) made up the remainder of patients. Over three-fourths of caregivers were the spouse or partner of the patient (n = 39, 78%).

**Table 2:** Participants’ self-reported background characteristics at baseline (n = 50).

Measure	n (%) or mean (SD), range
Age	54.4 (16.6), 20–80
Gender	
Female	19 (38.0)
Male	31 (62.0)
Ethnicity	
Hispanic or Latino	4 (8.0)
Not Hispanic or Latino	41 (82.0)
Missing	5 (10.0)
Race^a^	
White	32 (64.0)
Black	3 (6.0)
Asian	4 (8.0)
Native Hawaiian or Pacific Islander	1 (2.0)
Multiracial	4 (8.0)
Other	3 (6.0)
Missing	3 (6.0)
Educational Attainment	
Less than 9th grade	1 (2.0)
High school/GED	4 (8.0)
Some college, no degree	18 (36.0)
Associate’s degree	2 (4.0)
Bachelor’s degree	15 (30.0)
Master’s degree	8 (16.0)
Doctorate or other professional degree	2 (4.0)
Employment Status	
Unemployed	7 (14.0)
Disability leave	1 (2.0)
Homemaker	1 (2.0)
Retired	14 (28.0)
Self-employed	4 (8.0)
Student	3 (6.0)
Working part-time	2 (4.0)
Working full-time	18 (36.0)
CHLT-6	
Likely limited literacy	1 (2.0)
Likely adequate literacy	48 (96.0)
Missing	1 (2.0)
Relationship to Patient	
Spouse/partner	39 (78.0)
Parent	3 (6.0)
Child	5 (10.0)
Grandchild	1 (2.0)
Other relative	1 (2.0)
None-relative/friend	1 (2.0)
Patient’s Cancer Type	
Breast	17 (34.0)
Gynecological	12 (24.0)
Gastrointestinal	12 (24.0)
Thoracic	6 (12.0)
Other	3 (6.0)

SD = standard deviation

^a^ We did not have any participants who identified as American Indian or Alaska Native.

### Mobile app rating scales

The Day-28 in-app MARS items and the SUS scale showed that caregivers, overall, rated the TOGETHERCare app positively (**Table 3**). Both the mean and the median are presented in Table 3 to help describe the shape of the distributions. Because the mean and median are very similar, this demonstrates that the distributions are close to symmetrical. Median MARS ratings for app functionality (questions 1–4) were 4.5–5.0, at the highest end of the scale. The mean for these MARS questions ranged from 4.3 to 4.6. When combined, the MARS Functionality sub-score had a mean of 4.5 (SD 0.6) and median of 4.8. The median score for the MARS question on visual appeal (question 5) was 3.0, and the mean was 3.3 (SD 0.9). The raw total SUS score, usability sub-score and learnability sub-score translated into percentile rankings within the “excellent” range [59]. For the NPS administered via the app, “promoters” outnumbered “detractors” (4.2 percentage point difference). Results were stable for the in-app scores across the study period, so we present the final survey data in Table 3.

**Table 3:** Summary of the end of study in-app ratings^a^ (n = 50).

Selected MARS Questions	Mean (SD), Median
1. How accurately/fast do the app features (functions) and components (buttons/menus) work?	4.3 (0.8), 4.5
2. How easy is it to learn how to use the app; how clear are the menu labels/icons and instructions?	4.4 (0.9), 5.0
3. Is moving between screens logical/accurate/ appropriate/uninterrupted; are all necessary screen links present?	4.6 (0.8), 5.0
4. Are interactions (taps/swipes/pinches/scrolls) consistent and intuitive across all components/screens?	4.6 (0.7), 5.0
MARS functionality sub-score (sum of questions 1–4)	4.5 (0.6), 4.8
5. How good does the app look?	3.3 (0.9), 3.0
**SUS 10**	mean (SD), median
Total SUS score	83.3 (14.2), 87.5
Usability sub-score	80.9 (15.7), 84.4
Learnability raw sub-score	93.2 (12.6), 100.0
Total SUS score percentile rank [59]	90–95
**NPS** ^**b**^	n (%)
0–6 (Detractors)	17 (35.4)
7–8 (Passives)	12 (25.0)
9–10 (Promoters)	19 (39.6)
Promoters %—Detractors %	4.2

MARS = Mobile App Rating Scale; NPS = Net Promoter Score; SD = standard deviation; SUS = System Usability Scale

^a^ All final surveys were included in the statistics, even those from participants who completed them past the active study period of 28 days.

^b^ Two participants did not respond to the NPS. Therefore, the denominator for the NPS calculations was 48.

### Semi-structured interviews

The interview results suggest that over the course of the study period users were generally positive about the app’s ease of use, and many found the navigation intuitive. Caregivers found the app helpful in raising their awareness about the patient, as well as about their own well-being. Caregivers noted that they wanted feedback from the app to better understand the progress of the patients, or if there were symptoms that should cause them concern. While some felt more graphical features would be helpful, caregivers were pleased by its simplicity. Caregivers suggested survey modifications and enhancements that would be helpful in better responding to a few of the questions, and they wanted more notifications linked to awaiting tasks. **Table 4** further describes the themes and concepts and provides selected comments.

**Table 4:** TOGETHERCare app: qualitative analysis themes with illustrative comments.

Theme	Concept	Selected Comments
1. Easy to use	A majority of the caregivers said that the app was easy to use and intuitive.	“It’s very straightforward. It’s simple.” “Yeah. So the app being easy to use, I would agree. I just appreciate that it’s simple.” “I think it’s very self-explanatory. The layout is simplified. It’s intuitive. Yeah, it was very easy to figure out.” “It’s simple. It’s straightforward. Oh. It’s just very intuitive.”
2. Helpful, but should provide feedback	Most caregivers found the app useful for gauging how the patient was doing, and as a reminder to check on the patient for various things. Caregivers suggested the app could be improved by adding feedback, so that they could gauge the progress of the patient, or if there were symptoms that should cause them concern. Caregivers were appreciative of the fact that the app asked about them and not just the patient.	“Well, I think, gosh, if nothing else, it forces you to be communicating and gauging how someone else is doing… And if it helps with research, I think it’d be—gosh, I would recommend it.” “I’m not sure I would actually recommend it to somebody. They wouldn’t get anything out of it. Because I’m giving you information, but you guys aren’t really giving us any information back. There’s nothing coming back to me.” “… there was no feedback. It was just sort of giving information…” “It’s interesting knowing that my feedback matters to my partner’s care. And that it makes me conscious of my own feelings and my taking care of myself or not taking care of myself is an important aspect of being present with your partner, and not just focusing on just their health, but my own mental, physical.”
3. Survey questions, frequency and timing need enhancement as well as modifications	Caregivers offered several suggestions based on their experience with the app, such as a wider scale for the responses, more specific questions and less general questions, and timing the frequency of the surveys to the stage of treatment.	“So when I read the question, it’s like, what do you really want me to answer? How concerned am I today, or? I’m always concerned about her. And so I think if it were worded in such a way to say, "Do you have concerns with her health today more so than before?" Something to that effect.” “Has the other person taking their medications today right? Easy question. The problem is it’s purely a yes or no answer. Have they taken their meds today and if a person has to take medications at multiple times per day and this is one of those? How do I answer that question? They did take meds today, but they missed some of them, So what are they looking for in that answer for that question?”
4. Add more functionality as well as enhanced visuals to the app	Caregivers suggested that the app interface needed more color and features, but at the same time they did not consider a plain interface to be a negative feature.	“…I think it’s better to be a little bit more appealing, a little bit more, I don’t know, if decorative is the right word, but it felt very sterile, very clinical.”
5. Notifications/Technical issues	Like the patients, caregivers also suggested that the app will be more attractive as soon as issues with reminders, notifications, and other technical difficulties are resolved.	“am I going to get a notification? Or do I just go in there every other day or whatever and look?” “The biggest problem I have is that one-half to two-thirds of the time, I will open up the app and I get an error screen.” “I really benefit from push notifications, especially there’s so much going on that from my [inaudible] are very good for me.”

During the final semi-structured interviews after at least 28 days of use, interviewers presented revisions to the app that addressed the feedback, including themes, that we had received over the usage period. Interviewers then asked caregivers to provide the NPS considering these revisions. Thus, NPS interview scores were tabulated separately from the in-app NPS. The NPS score collected from the Day 7 interviews was 22; 26% were detractors and 48% were promoters. The NPS score collected from the Day 28 interviews was 30; 24% were detractors and 54% were promoters. We did not compare in-app NPS to interviewer NPS because of the difference in mode of administration and presentation of improved app functionality based on caregiver feedback and input.

## Discussion

The objective of this study was to test the usability of a novel app designed for informal caregivers of adult cancer patients receiving intravenous chemo- or immunotherapy. The MARS items and the SUS scale showed that caregivers, overall, rated the TOGETHERCare app very positively and that this was stable over the 28-day period. The SUS scores were in the “excellent” range, indicating that people were able to figure out how to use the app, and that there were no serious usability issues. The NPS also indicated that more respondents would promote the app than those who would not recommend the app.

The analysis of the semi-structured interviews indicated that the app was easy to use and helpful. Caregivers provided suggestions for enhancing the survey questions, visuals, and functionality, such as linking notifications to incomplete app tasks and providing feedback, especially about the progress of their patient. Because this study was designed primarily to determine usability from the caregiver’s standpoint, data collected by the app was not sent to the clinical care team or provided back to the user in the form of charts, graphs, or other compilations. However, it is technically feasible to provide bidirectional interaction with EHR systems.

Despite caregiver interest in using online and mobile tools [71], few apps have been developed with the informal caregiver in mind [72,73]. A recent search on PubMed found no articles on applications for caregivers to report adult patient outcomes. Research is also lacking on caregiver health and psychosocial needs, and there has been insufficient testing of technology interventions that focus on caregiver health and psychosocial needs [74,75]. A recent review of online interventions to support cancer caregivers concludes that they are still in the formative stage; more research is needed to assess the impact on caregiver health [76]. Additionally, users are not involved in the development of most mobile health applications, despite evidence that input from direct users results in a more successful mobile tool [77,78]. Indeed, based upon the NPS from the final semi-structured interviews, this study reinforces the importance of gathering and incorporating participant feedback. Our study is one of the first to evaluate the usability of an app-based program that allows cancer caregivers to report their own health status as well as that of their patients’ including specific symptoms.

### Future directions

Future work in a larger clinical sample will include providing survey data completed by caregivers to their own providers within the clinical workflow. We expect, when it is medically acceptable, to provide visualizations of the survey responses over time for users to view so that caregivers can be aware of their well-being and be provided with appropriate resources. We plan to further integrate with EHR systems and test the app’s impact on specific health outcomes in a randomized controlled trial.

### Limitations

First, we included only participants who spoke English because the app was only offered in English. Offering the app in other languages would require cultural adaptation, and we plan on developing these after completing efficacy studies. Second, only participants who owned an iPhone were eligible to participate. Because our usability testing and other results (to be published separately) were positive, an Android version of TOGETHERCare, has now been developed. Finally, due to the usability testing nature of this study, and per the IRB recommendations, the collected data were not relayed to the participating patients’ or caregivers’ providers. One exception was that the IRB wanted the research associate to be automatically alerted to survey scores for mental health measures from the PROMIS Global Health scale that exceeded a predetermined threshold. If that occurred, the system was set-up to alert the research associate by email so appropriate next steps could be conducted following KPNC policies.

### Conclusions

TOGETHERCare is the first mobile app developed specifically to capture adult cancer patient outcomes from the informal caregiver’s perspective. In addition, it tracks the caregiver’s own health and wellbeing, which can impact the patient. Caregivers’ willingness to complete frequent surveys about their patients and themselves is promising, but additional research is needed to determine if the app can be used to improve outcomes. In addition to monitoring the physical and mental health of the caregiver, future versions of this app could potentially provide important clinical information that could inform patient care in meaningful ways, including by reducing emergency room visits or improving care.
